# Self-Perception of Body Weight in Schoolchildren in Northeastern Mexico

**DOI:** 10.3390/ijerph192214779

**Published:** 2022-11-10

**Authors:** María Natividad Ávila-Ortiz, Ana Elisa Castro-Sánchez, Georgina Mayela Núñez-Rocha, Andrea Elizabeth Flores-Sias, Adriana Zambrano-Moreno, Verónica López-Guevara

**Affiliations:** Universidad Autónoma de Nuevo León, Facultad de Salud Pública y Nutrición, Nuevo Leon 66455, Mexico

**Keywords:** children, perception of weight status, scale, sensitivity, specificity

## Abstract

Mexican schoolchildren are among the individuals most affected by obesity in the world. It has been observed that body-image dissatisfaction has increased in children. We evaluated their body weight perception and its relationship with actual weight; we compared this variable on three different scales according to age and sex and determined the sensitivity and specificity of these scales. This cross-sectional study was conducted with students from public and private schools in Northeastern Mexico. Boys and girls aged 6–12 years (*n* = 533) were included in this study. To assess the body weight perception, the following scales were used: (A) Collins (figure rating scale), (B) Eckstein (parents’ perceptions of their child’s weight and health scale), and (C) Truby and Paxton (children’s body image scale). Agreement was evaluated using the Cohen’s kappa test, determining the sensitivity and specificity. Girls and children aged 10–12 years were more likely to perceive themselves adequately (their self-perception corresponds to the figure that indicates their weight status). The children showed increased body-image distortion in the three scales. In terms of sensitivity and specificity, children with overweight or obesity were more precisely identified in scale A, whereas a healthy weight was more clearly identified in scale C.

## 1. Introduction

Mexico is one of the leading countries in childhood obesity. This problem has a higher prevalence in the northern states of the country and in the urban communities. The most recent reports show that one in three children was affected by obesity and that one in two of those children born from 2010 is at risk of developing diabetes at some point in life [1].

Children affected by overweight or obesity are five times more likely than normal-weight children to be overweight or an adult with obesity [2]. In addition, numerous studies indicate that childhood obesity is associated with various health problems and has psychological and emotional consequences. Therefore, the quality of life of these children is lower than that of children diagnosed with cancer [3].

In 2016, obesity was declared a health emergency by Mexican health authorities [4]. According to the National Health and Nutrition Survey (*Encuesta Nacional de Salud y Nutrición*—ENSANUT) (2020), 38.2% of Mexican schoolchildren are affected either with obesity or some degree of overweight (18.6% vs. 19.6% respectively). Obesity is more prevalent in boys, and overweight is more prevalent in girls. The comparison of the 2020 ENSANUT with the surveys from other years (2018, 2012, 2006, and 1999) showed an upward trend in obesity in both boys and girls. In 2018, the number of children with overweight decreased by 1.8% and has remained the same since, at 17.7%. In the same year, the number of girls with overweight decreased by 1.8%, reaching a prevalence of 18.4%, but it had increased to 21.6% by 2020 [5].

Body weight perception plays a key role because it involves feelings, attitudes, and thoughts related to weight, size, shape, and appearance. Body weight perception is a predictor of body management and body-mass-index (BMI)-related behaviors, that is, the actual weight status [6,7]. Body weight misconception is a risk factor for disordered eating behaviors, which is another factor that could explain its association with obesity. Various authors [8,9,10,11] indicate that body-image dissatisfaction has increased in schoolchildren, who consequently seek quick or easy strategies to lose weight and initiate eating disorders. These disorders affect their health and limit the seeking of help from healthcare professionals. In addition, an erroneous perception can have further consequences on the life of a child, not only with respect to their health status but also through their social environment and their emotional well-being, which affect their lifestyle [12].

Some studies have demonstrated that the relationship between body weight perception and real body weight is relatively low [13,14]. Children and adolescents cannot accurately perceive their weight status. Previous studies conducted in other countries have concluded that children tend to represent themselves as figures that are thinner than they actually are, showing dissatisfaction with their real bodies [15,16]. In turn, other authors [17,18] have demonstrated that girls are more likely to perceive themselves as overweight when compared to boys, regardless of their BMIs.

In Mexico, studies on body weight perception have focused on the female and urban population of the country, as well as on adolescents and adults [19,20,21,22]. Few studies have been conducted with school-age children to assess their body perceptions. Therefore, this study investigates the body weight perceptions in school-age children based on the following three scales, chosen for their differences, similarities, validity, and reliability: (A) Collins (figure rating scale), which has been used in Mexico in studies on the father, mother, or guardian of the student, comparing the body weight perceptions between parents and children [23,24,25]; (B) Eckstein (parents’ perceptions of their child’s weight and health scale), which contains figures designed by graphic designer Scott Millard and has been applied in the United States of America and Turkey, but not to the Mexican population; (C) Truby and Paxton (children’s body image scale), which is used in countries such as Australia and Brazil and has been applied to children aged 9–11 years in a small sample of northern Mexico [26].

Considering the above, this study aimed to compare three scales to find the best at measuring self-perceived body weight, thereby determining their sensitivity and specificity.

## 2. Material and Methods

From October 2020 to June 2021, a cross-sectional study was conducted with 533 boys and girls aged 6–12 years who were enrolled and attended their lessons online at public or private primary schools of the state of Nuevo León. Boys and girls aged 6–12 years, whose parent(s) or tutor signed an informed consent form and who accepted to participate in this research, were included in the study. The exclusion criteria were having some physical or psychomotor disability that did not allow them to answer the questions and not having their parents’ consent or not giving personal consent. To find the possible candidates for the study, directors and teachers from different schools were contacted (convenience sampling) [27]. Participants were included in the study when the parents or guardians of a boy or girl aged between 6 and 12 years and attending primary school agreed to participate in this research.

The sample size was large enough for a 95% CI and a 5% margin of error, given that the prevalence results ranged from 52% (overestimated and underestimated perception on the Truby and Paxton scale) to 82% (overestimated and underestimated perception on the Collins scale). We estimated 99 children as the minimum sample size for each scale considering previous differences in the calculation with a power of 90% and an alpha of 0.05. We included 533 children; each answered the three surveys. A total of 620 children were invited to participate, 560 gave their consent, 550 children answered the scales, and 533 children were measured. The present study was approved by the research ethics committee of the School of Public Health and Nutrition, Autonomous University of Nuevo León (Universidad Autónoma de Nuevo León—UANL) (20-FASPYN-SO- 03.TP). The corresponding informed consent was provided by all the parents or tutors, informed assent was given by the participants, and the Helsinki declaration was respected.

### 2.1. Variables

#### 2.1.1. Anthropometric Variables

The measurements were taken in the children’s homes once the parents or guardians agreed with the most convenient day and time for this purpose while ensuring that all health measures and recommendations were met due to the COVID-19 pandemic. The children wore light clothing and no shoes. They were weighed on a scale (Seca 804) that is accurate to 0.1 kg. Height was measured using a stadiometer (Seca 206, precision of 0.1 cm). Based on these data, the BMI was calculated, and the percentiles were calculated from the growth curves designed by the Centers for Disease Control and Prevention (CDC), which considers BMI-for-age weight status categories and the corresponding percentiles: underweight (below the 5th percentile); normal weight (between the 5th and 85th percentiles); overweight (between the 85th and 95th percentiles); obesity (above the 95th percentile) [28].

#### 2.1.2. Perception of Weight Status

The body weight perception was determined using three scales (A, B, and C), for which we used seven silhouettes or body figures with the same height but ranging from thinner to higher weight. Scale A was developed by Collins (1991) [29] and has seven human figures, whose dimensions gradually increase from thin to higher-weight silhouettes, so that each silhouette is assigned a specific BMI and classified as (1) severely underweight, (2) moderately underweight, (3) slightly underweight, (4) normal weight, (5) slightly overweight, (6) moderately overweight or (7) obesity. With a criterion-related validity between 0.36 and 0.37, and a reliability of r = 0.38–0.71 according to the test–retest method, this instrument was developed in the United States in 1991 and has been applied in school populations from different countries, including the Mexican population.

Scale B was developed by Eckstein et al. [30] and contains seven sketches, designed by the graphic artist Scott Millard, divided by age group (6–9 and 10–13 years) (order: left-end image = highest weight; right-end image = lowest weight). The central image (4) represents a child in the 50th BMI percentile. The first two figures on the left side, the three in the middle, and the penultimate and last figures on the right side were considered as low weight, normal weight, overweight, and obesity. The test–retest reliability of body weight perception was assessed on two separate occasions for 1 to 3 days. The percent agreement between the test and retest responses for sketch selection was 91.7% (two participants chose the next heaviest sketch upon retest).

Scale C, the children’s body image scale (CBIS) [31], was developed in Australia, in 2002, and was applied in British, Brazilian, and Mexican schools. It consists of seven figures based on the photographs of boys and girls at different BMI percentiles (3, 10, 25, 50, 75, 90, and 97). The instrument demonstrates good test–retest reliability for body figure perception (r = 0.85 for girls and r = 0.76 for boys, *p* < 0.001).

The three scales were applied to each participant to evaluate the weight perceptions of children. For this purpose, they were asked to answer the following question: which of these figures do you think you resemble? All the instruments used had seven figures, which allowed recoding of the values to build the following four categories: low weight, normal weight, overweight, and obesity, according to the CDC classification [28]. Under- or overestimation was assessed from the negative or positive difference between self-perception and BMI; a difference equal to 0 was interpreted as adequate perception.

Sensitivity was defined as the ability of the scale to classify schoolchildren as overweight or with obesity as such. Specificity was defined as the capacity of the data to classify a person as not overweight or with obesity who is actually neither one nor the other. To complement the diagnostic validity test, we determined the positive predictive value (PPV) of the reported data (the probability that a subject is overweight or with obesity if a positive result has been obtained based on the reported data), and the negative predictive value (NPV)—the probability that a non-obese subject is classified as such by the reported data [32].

### 2.2. Procedure

With the support of trained nutrition staff, the parents of children who met the inclusion criteria were contacted by phone. The objectives of the study were explained to them, and permission to apply the three scales through video calls was requested. The parents who accepted the invitation were sent the informed consent form electronically (by mail or WhatsApp) and asked to send the signed form back in the same manner. Subsequently, the following steps were followed: (a) home visit to take anthropometric measurements; each meeting took approximately 10 min; (b) appointment for a video call to apply the scales; (c) before starting the questionnaire, the consent of the minors was confirmed by asking the children whether they were willing to participate in the study.

#### Statistical Analysis

The descriptive statistics were analyzed by sex, age, and school grade. The relationship between the measured categories and perceived BMI was studied to assess the accuracy of body weight perception using the χ^2^ and Fisher’s exact tests. To assess the agreement between the categories of measured and perceived BMI, the Cohen’s kappa coefficient was calculated and analyzed based on Landis and Koch [32], considering κ ≤ 0.20 as slight, 0.21 ≤ κ ≤ 0.40 as fair, 0.41 ≤ κ ≤ 0.60 as moderate, 0.61 ≤ κ ≤ 0.80 as substantial, and 0.81 ≤ κ ≤ 1.00 as almost perfect. The significance level was set at *p* < 0.05. In addition, we calculated the sensitivity, specificity, and positive (PPV) and negative (NPV) predictive values of the three scales in classifying overweight/obesity and normal weight according to the measured data. The IBM SPSS Statistics Version 25 program was used. The reliability of the scales was: scale A α = 0.59; scale B α = 0.63; scale C α = 0.54. None of the scales resulted in a normal distribution, and therefore, it was decided to use nonparametric tests.

## 3. Results

Table 1 outlines the descriptive characteristics of the sample. We investigated boys and girls from Northeastern Mexico, aged 6–12 years, who were attending primary school online. The sample included 533 children, namely, 276 (51.8%) girls and 257 (48.2%) boys. The mean age was 8.5 years (8.5 and 8.6 years for boys and girls, respectively). The following age groups were considered: 6–9 years (65.3%) and 10–12 years (34.7%). According to the BMI calculated based on weight and height and the corresponding CDC percentile, 6.8% children were underweight and 42.1% children were overweight.

Table 2 presents the association between the measured BMI and body weight perception in the total sample (*n* = 533). Scales B and C show statistical significance between measured BMI and body weight perception (*p*= 0.000). In scale A, no association was found between overweight and the perception of body image (χ2 = 5.592, df = 2, *p* > 0.05). Another analysis was performed to assess the association of the measured BMI and distorted body weight; the variable body weight perception was recoded, grouping the children who did present distortion in their perceptions (children with overestimated and underestimated perceptions) and those who did not present distortion (children with adequate perception). In scale A, no association was found between distorted body weight and overweight (χ2 = 2.830, df= 1, *p* > 0.05), and in scales B and C, a statistically significant association was observed (*p* = 0.000). For scale B, underweight was not related to distorted body weight (χ2 = 0.488, df = 1, *p* > 0.05). However, for scales A and C, a significant association was verified. When relating normal weight and obesity with weight distortion, these were significant (*p* = 0.000) in the three scales.

Table 3 presents the sex differences between the measured and perceived BMI by scale. Both sexes presented a higher percentage of adequate body weight perception by scale C (46.3%) (44.6% boys and 55.4% girls). The scale with the highest percentage of underestimation was scale A (68.3%), whereas scale B showed the highest percentage of overestimation (7.7%). Scales A and C showed significant sex differences between the measured BMI and body weight perception. However, when broken down by measured BMI, only scale A showed significant differences in the children’s normal weight (χ^2^ = 16.64, df  =  2, *p* = 0.000).

Table 4 presents the age differences by scale; a higher percentage of adequate body weight perception was found when using scale C in both age groups, that is, 6–9 years and 10–12 years; the highest overestimation was observed for scale B, whereas scale A showed the highest underestimation in both age groups. No association was found between the body weight perception and age with the three scales; however, when breaking down the data by measured BMI, normal weight schoolchildren showed significant associations in both scale B (χ^2^ = 7.23, df  =  2, *p* < 0.05) and scale C (χ^2^ = 14.45, df  =  2, *p* < 0.05).

The degree of agreement between the measured and perceived BMI was analyzed using Cohen’s kappa coefficient (Table 5). This identified significant associations in the three scales. Cohen’s kappa coefficient was 0.011 in scale A; 0.032 in scale B; 0.44, the highest, in scale C. These indices are considered as poor according to the normative values of this test.

Table 6 outlines the sensitivity, specificity, and the positive (PPV) and negative (NPV) predictive values of the three scales, which reflect the validity values of using the instruments to classify schoolchildren as overweight or with obesity. Scale A showed the highest sensitivity (9.87%), along with 69.34% specificity, 20.75% PPV, and 48.59% NPV upon comparison of the three scales.

By sex, scale A showed a sensitivity and specificity of 11.63% and 60.55% for boys and 7.45% and 75.15% for girls, respectively. The sensitivity and PPV were higher for boys than for girls. Scale B showed a sensitivity and specificity of 8.53% and 23.85% for boys and 7.45% and 34.55% for girls, respectively. The specificity and NPV were higher for girls. Scale C showed a sensitivity and specificity of 1.28% and 11.93% for boys and 6.38% and 17.58% for girls, respectively. The sensitivity, specificity, and NPV were higher for girls than boys (Table 6).

As outlined in Table 7, scale C had high sensitivity (84.67%) by adequately classifying students with normal weight, whereas its specificity was 81.08%. By sex, scale C showed a sensitivity and specificity of 88.07% and 96.62% for boys and 82.42% and 90.99% for girls, respectively. The values of sensitivity, specificity, PPV, and NPV of scale C were higher for boys than girls.

Scale B showed a sensitivity and specificity of 76.15% and 89.19% for boys and 65.45% and 86.49% for girls, respectively. The sensitivity, specificity, and NPV values of scale B were higher for boys than girls. Scale A showed the lowest sensitivity and specificity: 39.45 and 81.08% for boys and 24.85% and 81.08% for girls, respectively.

## 4. Discussion

The purposes of this study were to evaluate the body weight perception and its relationship with the BMI measured and to compare this variable using three different scales according to age and sex. Differences were found between the measured BMI and body weight perception in the three scales. The high percentage of participants with distorted body image stood out. It is notable that most children with normal weight were perceived as overweight or underweight, and that those affected by obesity considered that they were only overweight. Scale A showed the highest percentage of inadequate body weight perception (75.1%), a result that differs from a study conducted in Asia using this same scale, in which a higher percentage of body image distortion (82%) was found. Body dissatisfaction was also associated with weight status: for overweight and with obesity, children were more likely to select thinner ideal body sizes than healthy-weight children [33]. In this study, 58.6% of children perceived their body weight erroneously when using scale B; when mothers used scale B to perceive the body weight of their children, the results of distorted perception were lower (43.1%) [34].

With scale C, approximately 6 of 10 children perceived their image inadequately. In this regard, a study conducted with Hispanic children aged 8–11 years in a rural community in New Mexico, USA, using the same scale, found similar results: 51.7% of children failed to perceive themselves adequately [15]. In another study [26], the degree of precision with which schoolchildren perceived their body image was higher than that reported in this study. The sample of this study was smaller, and the scale was only applied to children between 8 and 13 years of age. Moreover, of the three scales used, scale C showed the highest percentage of adequate perception.

When comparing the measured BMI, body weight perception, and sex between scales A and C, significant sex differences were found. In scale A, boys were observed to overestimate their body image when compared to girls. A study conducted in China indicated contrasting results: boys perceiving themselves as too thin and girls perceiving themselves as too heavy [17]. Girls wanted to be slimmer; the explanation may be that the media has promoted the ideal of a slim female body. However, in a study performed using the same scale, the authors concluded that boys more commonly have an inadequate perception due to the overestimation of their body image and that being male increases the risk of presenting body image distortion by two-fold [18].

Scale C resulted in a more overestimated perception and a higher percentage of girls perceiving themselves adequately, in line with a study conducted in the Netherlands, which indicated that girls are more likely to perceive themselves as normal weight or overweight [35]. Various studies indicate that girls and women have a higher prevalence of distorted body image [36,37,38,39]. Therefore, the number of cases of excessive body dissatisfaction or concern over the years is currently increasing in both boys and girls, who idealize and perceive themselves as thinner and expect to have slimmer bodies [13,40].

The 6–9 age group demonstrated more underestimation and overestimation through the three scales, albeit with no significant association between the measured BMI, body perception, or age. Children aged 10–12 years tend to have more adequate self-perception, a result that matches findings reported in a study conducted in Colombia in which the authors highlighted that 11-years-old children presented the best body image perception [18]. In this regard, current scientific literature on the impact of obesity on body image indicates that, from the age of 11 years, boys and girls are dissatisfied with their figures and with the ensuing feelings, and that, at least on some occasions, they control food intake to avoid gaining weight [41]. Although the desire to lose weight arises from an early age, a greater number of preadolescent girls go to weight loss clinics, citing body dissatisfaction; the studies also indicate that age corresponds to the first stage of the transition toward adolescence, with important physical changes. Furthermore, they have already acquired the cultural norms and stereotypes underlying attractiveness [42].

The results of this study showed that the global prevalence of accurate body weight perception in the three scales ranges from 24.9 to 46.1%, which reflects a poor agreement index of between 0.011 and 0.044, according to the classification of Landis and Koch. This finding makes sense by showing differences between the measured BMI and body weight perception. Underweight children tend to overestimate their weight, whereas those with normal weight, overweight, and obesity tend to underestimate their weight, particularly those in the last two categories. In China, a study conducted with a representative sample of children at the regional level using scale A found a deficient consistency of BMI and weight perception, a result that matches that presented in this study [13]. In contrast, the results of a study performed in northern Mexico using scale C reflected an acceptable agreement index with an adequate perception of 59.4% [26].

According to the literature, no studies conducted with children have reported the sensitivity and specificity of the scales. However, a comparison of our results with those of other studies that have assessed body weight perception—but with the parents of the children, such as a study in which scale A was used—showed higher sensitivity for the other studies than that reported in the present study. Nevertheless, the specificity was approximately the same (60%) in our study and the aforementioned one [43]. Another study found that the sensitivity and specificity of scale B for evaluating the parents’ perceptions of their children with overweight by images were 70 and 84%, respectively, percentages well above those found in our study [30]. In this regard, the heaviest and shortest schoolchildren contributed the most to such inaccuracy.

This study had some limitations. First, it is possible that the self-perception of body weight was overestimated; the children could have provided socially acceptable answers. However, we consider this bias low as the trained nutritional professionals administered the questionnaire, and anonymity was guaranteed at the time of its application. Second, this study only involved children aged 6 to 12 years old. Therefore, the results may include a selection bias, which might only represent some of the northeastern Mexico schoolchildren. Third, the research was cross-sectional; therefore, causality could not be determined. We only assessed the association between BMI and the body weight perception by age and sex. Future studies should investigate the associations between the place of residence, school grade, eating habits, weight dissatisfaction and concerns, physical activity, and psychological symptoms.

Despite these limitations, our study had advantages, namely, (a) comparing different scales to measure the body weight perception (previous studies mainly focused on one scale and sometimes only on the perception of the mother, father, or guardian; in addition, few studies have been conducted with children in the country); (b) although the data were collected during a pandemic, BMI was estimated by nutritional professionals measuring the height and weight, and not based on the self-reported data; (c) we analyzed the association between BMI and body weight perception and emphasized the importance of body weight perception (misperception is detrimental to children’s health and normal growth); (d) the most relevant aspect of this study is that the results provide useful information for policymakers to make evidence-based decisions for promoting healthy growth in children.

### Implications for Research and Practice

Overweight and childhood obesity continue to increase, especially in our country; despite the various strategies to reduce them, excess body fat in children is being normalized and minimized by pointing out that it is an aesthetic problem. Our findings suggest that self-perceived body image in school-age children may play an essential role in excessive weight gain, thus requiring various strategies to design and implement successful public health interventions. In this sense, scales such as those used in this study can show different dimensions of body self-perception and nutritional status, which should be contemplated when studying the multiple population sectors and the proposed objectives. In addition, triangulation through instruments could be considered; this contribution could improve understanding and provide accurate information about possible body dissatisfaction, which will allow a better understanding of body image perception and potentially impact childhood obesity. Given this, health professionals can play a central role by focusing on programs that promote a healthy lifestyle, a positive body image, the acceptance of the diversity of body sizes, and the prevention of eating disorders.

## 5. Conclusions

Girls and children in the 10–12-year-old group were more likely to perceive themselves adequately. In our study, with the three scales, the boys showed more distortion of body weight perception when compared to girls, who evaluated their weight better than the boys. The overall prevalence of children who adequately perceived their body weight was higher when using scale C. In terms of the sensitivity and specificity, scale A is more precise with overweight and obesity figures, whereas healthy weight seems to be more clearly observable using scale C.

Therefore, schools and parents should take specific steps to help boys improve their weight control and increase the awareness of their weight categories, thus helping to improve body image accuracy and in turn promoting reasonable dietary habits and physical activities. The criteria for evaluating the body image perception in children should also be homogenized for international and regional comparisons.

## Figures and Tables

**Table 1:** Descriptive characteristics of the sample (*n* = 533).

	Female	Male	Total
Age (Mean, SD)	8.5 (1.80)	8.6 (1.79)	8.5 (1.79)
Weight (Mean, SD)	32.3 (11.32)	35.3 (12.26)	33.7 (11.87)
Height (Mean, SD)	127.3(28.76)	128.6 (30.53)	128(29.81)
Age (years)			
6–9	67%	64.5%	65.8%
10–12	33%	35.5%	34.2%
Weight status			
Underweight	6.2%	7.4%	6.8%
Normal weight	59.8%	42.4%	51.4%
Overweight	15.2%	19.8%	17.4%
Obesity	18.8%	30.4%	24.4%
Type of school			
Public	69.9%	72.8%	71.3%
Private	30.1%	27.2%	28.7%

Mean/Standard deviation.

**Table 2:** Association between the measured BMI and body weight perception.

Measured BMI	Adequate	Overestimated	Underestimated	*p*-Value *
SCALE A				
Underweight	75%	25%	0	0.000 **
Normal weight	30.7%	8.8%	60.6%	0.002 *
Overweight	18.3%	3.2%	78.5%	0.055
Obesity	3.8%	0	96.2%	0.000 **
SCALE B				
Underweight	36.1%	63.9%	0	0.000 **
Normal weight	69.7%	5.8%	24.5%	0.000 **
Overweight	9.7%	2.2%	88.2%	0.000 **
Obesity	6.9%	0	93.1%	0.000 **
SCALE C				
Underweight	22.2%	72.2%	5.6%	0.000 **
Normal weight	84.7%	2.9%	12.4%	0.000 **
Overweight	2.2%	1.1%	96.8%	0.000 **
Obesity	3.8%	0	9.2%	0.000 **

Note: Fisher’s exact test. * *p*-value < 0.05 (significantly associated). ** *p*-value < 0.001 (highly significantly associated).

**Table 3:** Sex differences between the measured BMI and body weight perception by scale.

Measured BMI	Male			Female			*p*-Value *
	Adequate	Overestimated	Underestimated	Adequate	Overestimated	Underestimated	
SCALE A							
Underweight	68.4%	3.6%	0	82.4%	17.6%	0	0.45
Normal weight	39.4%	14.7%	45.9%	24.8%	4.8%	70.3%	0.00 **
Overweight	23.5%	3.9%	72.5%	11.9%	2.4%	85.7%	0.35
Obesity	3.8%	0	96.2%	3.8%	0	96.2%	1.00
SCALE B							
Underweight	26.3%	73.7%	0	47.1%	52.9%	0	0.29
Normal weight	76.1%	5.5%	18.3%	65.5%	6.1%	28.5%	0.14
Overweight	11.8%	2%	86.3%	7.1%	2.4%	90.5%	0.75
Obesity	6.4%	0	93.6%	8%	0	92.3%	1.00
SCALE C							
Underweight	21.1%	68.4%	10.5%	23.5%	76.5%	0	0.60
Normal weight	88.1%	3.7%	8.3%	82.4%	2.4%	15.2%	0.21
Overweight	0	0	100%	4.8%	2.4%	92.9%	0.08
Obesity	1.3%	0	98.7%	7.7%	0	92.3%	0.15

Note: Fisher’s exact test. * *p*-value < 0.05 (significantly associated). ** *p*-value < 0.001 (highly significantly associated).

**Table 4:** Age differences between the measured BMI and body weight perception by scale.

Measured BMI	6–9 Years			10–12 Years			*p*-Value *
	Adequate	Overestimated	Underestimated	Adequate	Overestimated	Underestimated	
SCALE A							
Underweight	73.1%	26.9%	0	80%	20%		0.51
Normal weight	29.6%	8.9%	61.5%	33.3%	8.9%	57.8%	0.88
Overweight	11.3%	3.8%	84.9%	27.5%	2.5%	70%	0.09
Obesity	5.6%	0	94.4%	0	0	100%	0.32
SCALE B							
Underweight	46.2%	53.8%		10%	90%	0	0.06
Normal weight	64.2%	6.7%	29.1%	80%	4.4%	15.8%	0.02 *
Overweight	7.5%	3.8%	88.7%	12.8%	0	88.7%	0.48
Obesity	5.6%	0	94.7%	10%	0	90%	0.45
SCALE C							
Underweight	30.8%	65.4%	3.8%	0	90%	10%	0.11
Normal weight	78.8%	3.9%	17.3%	95.8%	1.1%	3.2%	0.00 **
Overweight	3.8%	1.9%	94.3%	0	0	100%	0.50
Obesity	3.4%	0	96.7%	5%	0	95%	0.64

Note: Fisher’s exact test. * *p*-value < 0.05 (significantly associated) ** *p*-value < 0.001 (highly significantly associated).

**Table 5:** Consistency analysis between the measured BMI and perceived BMI by scale.

Measured BMI	PERCEIVED BMI				Agreement	Kappa	*p*-Value *
	Underweight	Normal Weight	Overweight	Obesity			
SCALE A							
Underweight	75%	22.2%	2.8%	0		0.01	0.00 **
Normal weight	60.6%	30.7%	8%	0.7%	24.9		
Overweight	34.4%	44.1%	18.3%	3.2%			
Obesity	10%	35.4%	50.8%	3.8%			
SCALE B							
Underweight	36.1%	63.9%	0	0	41.4	0.03	0.00 **
Normal weight	24.8%	69.3%	4.7%	1.1%			
Overweight	15.1%	73.1%	9.7%	2.2%			
Obesity	5.4%	73.1%	14.6%	6.9%			
SCALE C							
Underweight	22.2%	77.8%	0	0	46.1	0.04	0.00 **
Normal weight	10.9%	84.3%	3.6%	1.1%			
Overweight	4.3%	92.5%	2.2%	1.1%			
Obesity	2.3%	79.2%	14.6%	3.8%			

Note: * *p*-value < 0.05 (significantly associated). ** *p*-value < 0.001 (highly significantly associated).

**Table 6:** Sensitivity and specificity of the scales for overweight and obesity.

SCALE A	Boys	Girls	Total
	%CI	%CI	%CI
Sensitivity	11.63 (5.71–17.55)	7.45 (1.61–13.29)	9.87 (5.73–14.00)
Specificity	60.55 (50.92–70.18)	75.15 (68.25–82.05)	69.34 (63.70–74.98)
PPV	25.86 (13.73–37.99)	14.58 (3.56–25.61)	20.75 (12.56–28.95)
NPV	36.67 (29.35–43.98)	58.77 (51.89–65.65)	48.59 (43.51–53.68)
SCALE B			
Sensitivity	8.53 (3.32–13.73)	7.45 (1.61–13.29)	8.07 (4.27–11.87)
Specificity	23.85 (15.39–32.31)	34.55 (26.99–42.10)	30.29 (24.67–35.92)
PPV	11.70 (4.67–18.73)	6.09 (1.28–10.89)	8.61 (4.57–12.66)
NPV	18.06 (11.43–24.69)	39.58 (31.25–47.92)	28.82 (23.41–34.22)
SCALE C			
Sensitivity	1.28 (0.00–4.42)	6.38 (0.91–11.86)	3.14 (0.63–5.65)
Specificity	11.93 (5.38–18.47)	17.58 (11.47–23.69)	15.33 (10.88–19.78)
PPV	7.49 (3.45–11.53)	4.23 (0.56–7.89)	2.93 (0.58–5.28)
NPV	14.44 (6.63–22.26)	24.79 (16.54–33.04)	16.28 (11.58–20.98)

Note: CI, confidence interval.

**Table 7:** Sensitivity and specificity of the scales for normal weight.

SCALE A	Boys	Girls	Total
	%CI	%CI	%CI
Sensitivity	39.45 (29.82–49.08)	24.85 (17.95–31.75)	30.66 (25.02–36.30)
Specificity	81.08 (74.43–87.73)	81.08 (73.34–88.82)	81.08 (76.12–86.04)
PPV	60.56 (48.49–72.64)	66.13 (53.54–78.72)	63.16 (54.58–71.73)
NPV	64.52 (57.37–71.66)	42.06 (35.21–48.90)	52.50 (47.48–57.52)
SCALE B			
Sensitivity	76.15 (67.69–84.61)	65.45 (57.90–73.01)	69.71 (64.08–75.33)
Specificity	89.19 (83.85–94.53)	86.49 (79.68–93.30)	88.03 (83.88–92.18)
PPV	83.84 (76.08–91.59)	87.80 (81.62–93.99)	86.04 (81.25–90.82)
NPV	83.54 (77.45–89.64)	62.75 (54.76–70.73)	73.31 (68.24–78.39)
SCALE C			
Sensitivity	88.07 (81.53–94.62)	82.42 (76.31–88.53)	84.67 (80.22–89.12)
Specificity	96.62 (93.37–99.87)	90.99 (85.21–96.77)	94.21(91.17–97.25)
PPV	95.05 (90.32–99.78)	93.15 (88.71–97.59)	93.93(90.75–97.11)
NPV	91.67 (87.01–96.32)	77.69 (70.15–85.23)	85.31(81.04–89.59)

Note: CI, confidence interval.

## Data Availability

Not applicable.
